# OLDER ADULT MISUSE OF OVER-THE-COUNTER MEDICATIONS: BENEFITS OF A NOVEL PHARMACY-BASED INTERVENTION

**DOI:** 10.1093/geroni/igad104.3570

**Published:** 2023-12-21

**Authors:** Jason Chladek, Aaron Gilson, Jamie Stone, Elin Lehnbom, Emily Hoffins, Maria Berbakov, Jukrin Moon, Michelle Chui

**Affiliations:** University of Wisconsin-Madison School of Pharmacy, Madison, Wisconsin, United States; University of Wisconsin-Madison, Madison, Wisconsin, United States; University of Wisconsin - Madison, Madison, Wisconsin, United States; UiT The Arctic University of Norway, Tromsø, Troms, Norway; University of Wisconsin-Madison, Madison, Wisconsin, United States; University of Wisconsin-Madison, Madison, Wisconsin, United States; University of Wisconsin-Madison School of Pharmacy, Madison, Wisconsin, United States; University of Wisconsin-Madison, Madison, Wisconsin, United States

## Abstract

Older adults’ inappropriate use of over-the-counter medications (OTCs) is prevalent and comprises: (1) Drug-Age misuse – medication risks due to advanced age, (2) Drug-Drug misuse – medication interactions, (3) Drug-Disease misuse – contraindications with health conditions, and (4) Drug-Label misuse – deviations from label instructions. Since pharmacies are ubiquitous sources of OTC accessibility and medication safety expertise, a structural redesign of pharmacy aisles (Senior SafeTM) was conceived to mitigate older adult OTC misuse. A central feature of Senior Safe is the use of Stop Signs for high-risk OTCs and Behind-the-Counter Signs (BTC) for particularly high-risk OTCs. Given the signage’s practical importance, this study’ principal aim was to determine whether Senior Safe reduced misuse of Stop Sign/BTC OTCs. Twenty pharmacies from a mid-Western healthcare system were matched and randomly allocated to either control or intervention groups, from which 288 older adults were recruited (ages 65-91). Participants’ reported OTC use for a symptom scenario (pain, sleep, cough/cold/allergy) was evaluated to determine whether it fulfilled the four misuse types. Misuse occurrences between intervention/control sites were compared using multivariate modeling. Drug-Age and Drug-Drug misuse frequencies were significantly lower in intervention sites (Coef.=-1.012, p=.004; Coef.=-1.702, p=.004, respectively), while Drug-Disease and Drug-Label misuse had too few occurrences in intervention sites for statistical comparisons. Also, adults aged 85+ had the greatest likelihood of all misuse types. Senior Safe shows substantial benefit reducing high-risk OTC misuse, which is important particularly for vulnerable adults ages 85-91. Future research should document intervention mechanisms contributing to lower older adult OTC misuse.

